# Systematic Medication Review in General Practice by an Interdisciplinary Team: A thorough but Laborious Method to Address Polypharmacy among Elderly Patients

**DOI:** 10.3390/pharmacy8020057

**Published:** 2020-03-31

**Authors:** Dagmar Abelone Dalin, Charlotte Vermehren, Anette Kobberø Jensen, Janne Unkerskov, Jon Trærup Andersen

**Affiliations:** 1 Department of Clinical Pharmacology, Copenhagen University Hospital Bispebjerg, Copenhagen DK-2400, Denmark; 2 Faculty of Health and Medical Sciences, University of Copenhagen, DK-2200 Copenhagen, Denmark; 3 The Hospital Pharmacy, the Capital Region of Denmark, DK-2730 Herlev, Denmark; 4 Quality in General Practice in the Capital Region of Denmark (KAP-H), DK-3400 Hillerød, Denmark

**Keywords:** medication errors, general practice, general practitioners, family practice, pharmacists, aged, aged 80 and over, polypharmacy, deprescriptions, healthcare professional, medication review, medicine review

## Abstract

Polypharmacy increases the risk of hospitalization but may be reduced by medication review. The study objective is to describe and evaluate a method for conducting medication review in general practice by an interdisciplinary medication team of pharmacists and physicians—in this case conducted by a team from the Department of Clinical Pharmacology—based on information concerning medication, diagnosis, relevant laboratory data and medical history supplied by the general practitioner. We discussed the medication review with the patients’ general practitioners and received feedback from them regarding acceptance rates of the recommended changes. Ninety-four patients with a total of 1471 prescriptions were included. A medication change was recommended for nearly half of the prescriptions (48%); at least one change of medication was recommended for all patients. The acceptance rate for recommended medication changes was 55%, corresponding to a mean of 4.2 accepted recommendations per patient. For 18% of all 1471 prescriptions, the general practitioner agreed either to discontinue (stop the medication completely) or reduce the dose of the medication. This method is thorough, but since it requires several healthcare professionals, it is rather time-consuming. There is a need to support medication review in general practice, but although this method may be too time consuming in most cases, it may nevertheless prove to be a useful tool managing the most complicated patients.

## 1. Introduction

Polypharmacy is a problem in modern medication and significantly increases the risk of hospitalization due to adverse drug reactions [1]. In particular, the elderly population is prone to adverse drug reactions, as they are frail and often suffer from multiple diseases for which they take various medications. Worldwide, adverse drug reactions account for 9% of all hospital admissions of patients older than 60 years of age [1]. In Denmark, similar results have been found for patients admitted to medical wards [2].

A medication review is a structured review of a patient’s medication that aims to optimize medications according to patient perspective, evidence and expenses. Medication reviews have been shown to reduce polypharmacy, which can reduce adverse drug reaction and hospital admissions [3]. Different healthcare professionals may focus on different aspects of medication, so an interdisciplinary team may be expected to be able to optimize the medication review [4]. Medication reviews are not performed routinely in general practice in Denmark, lack of time and skills in general practice being just two of the obstacles to doing them [5]. Possibly as a consequence of this, the literature tends to focus on medication reviews performed by healthcare professionals other than general practitioners (GPs). A thorough medication review is time-consuming—and for GPs in Denmark—not financially reimbursed directly. However, polypharmacy is an increasing problem; the care and treatment of chronic diseases have shifted from hospitals to general practice. These factors increase the need for medication reviews in general practice; it is important to continue developing a method that is feasible in general practice.

The purpose of this study is to describe a method for systematic medication review for elderly polypharmacy patients in general practice, conducted by a regional interdisciplinary medication team of pharmacists and physicians—and to evaluate the feasibility of the method.

## 2. Materials and Methods

### 2.1. General Practice in Denmark

In Denmark, GPs are self-employed and have a contract with the public funding authorities. Each citizen is affiliated with one general practice, where consultations and most treatments are free. GPs are financially reimbursed for the consultations and treatments from the Danish public funding authority. GPs prescribe medication which can be purchased by the citizen at a pharmacy.

### 2.2. The Setting

This project was a collaboration between the Department of Clinical Pharmacology, Copenhagen University Hospital Bispebjerg and Quality in General Practice in the Capital Region of Denmark (KAP-H) in 2017. KAP-H has ten GPs part-time employed as medical consultants who each regularly visit the GPs of the Capital Region of Denmark to support rational pharmacotherapy, which is defined as effective, safe, appropriate and economical medication.

We used this established organization to include GPs and patients throughout the Capital Region of Denmark. Approximately one third of the 620 general practices in the region had one visit by a medical consultant from KAP-H and were invited to participate in this project with a medication review of one patient.

### 2.3. Inclusion of Patients

We asked each of the GPs to include one complicated patient (aged ≥ 65 years using ≥ 6 medications) for the medication review study. The age of 65 years is commonly accepted as a threshold between younger and elderly patients [6,7]. The cut-off of six medications was arbitrarily chosen to ensure the study of patients with complex medication regimes. These medications could be prescription medicines or over the counter drugs but did not include vitamins. However, vitamins were still assessed during the medication review.

### 2.4. Design

This is an overview of the study design. The steps will be described in detail in the following sections.

**Figure 1:**
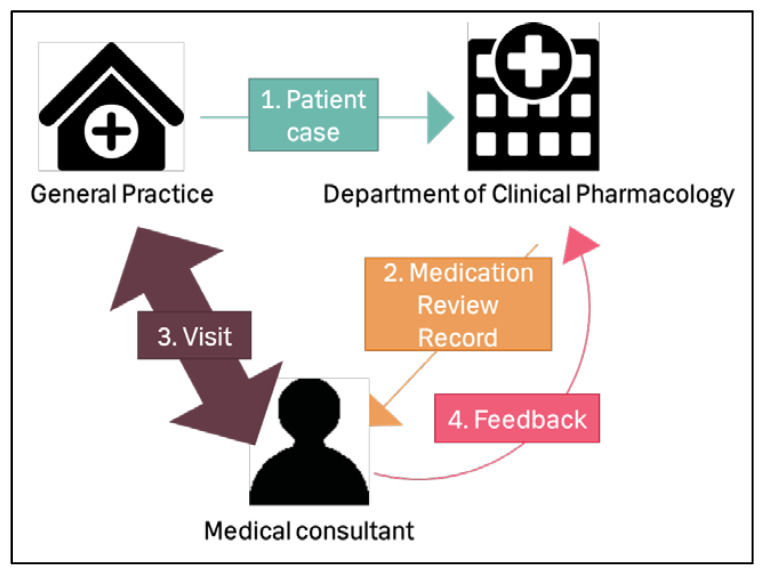
Study design. 1. The general practitioner (GP) sent a patient case to the Department of Clinical Pharmacology, who did the medication review. 2. Department of Clinical Pharmacology sent a record of the medication review to the medical consultant. 3. The medical consultant visited the GP and discussed the changes recommended in the record. 4. The medical consultant reported feedback from the GP to the Department of Clinical Pharmacology.

1. Each GP sent one patient case (consisting of the patient’s medical and health information) to the medication team at the Department of Clinical Pharmacology, Copenhagen University Hospital Bispebjerg (Figure 1). The medication team consisted of a group of pharmacists and physicians. Each medication review was conducted by one pharmacist and one physician from the medication team. An interdisciplinary medication team was chosen because we considered different healthcare professionals may find different medication-related problems [4]; this would improve the quality of the medication review. They registered the recommended changes for the medication in a medication review record.
2. This record was sent electronically to the medical consultant from KAP-H.
3. The medical consultant then visited the GP in person, and they discussed the medication review.
4. The medical consultant reported feedback electronically to the medication team about which of the recommended changes the GP accepted.

This study involved four healthcare professionals: the patient’s GP, the physician and pharmacist from the medication team at the Department of Clinical Pharmacology and the medical consultant from KAP-H.

### 2.5. Patient Case

We asked the GPs to include the following information in the patient case: a list of currently prescribed medications, including dose and indications, diagnoses, relevant laboratory data and medical history. The patient case was sent to the Department of Clinical Pharmacology at least 14 days before the visit from the medical consultant. We assumed that performing a medication review based on this information would be thorough and result in clinically relevant recommendations.

The laboratory data we deemed most relevant were renal function, as estimated by the glomerular filtration rate (eGFR), blood sugar measured as glycated hemoglobin (HbA1c), cholesterol measured as low-density lipoprotein (LDL) and blood pressure. We defined an eGFR ≥ 60 mL/min as normal. [8] HbA1c values above 48 mmol/mol are diagnostic of diabetes mellitus type 2. [9] The HbA1c goal-of-care value for antidiabetic treatment is individual for the patient on the basis of characteristics such as the patient’s age, risk of hypoglycemia, comorbidities and disease progression. [9] Hypertension was defined as blood pressure above 140/90 mmHg, but antihypertensive medication may be started at a lower threshold if some comorbidities are present. [10]

A complete case was defined as having either diagnoses or indications and renal function, as well as laboratory data for cholesterol, blood pressure and blood sugar, if relevant. Laboratory data were deemed relevant if the patient had a diagnosis of hypercholesterolemia, hypertension, diabetes mellitus or similar, or if medications had been used specifically to treat these diseases.

### 2.6. Medication Review

The medication review was built upon the patient case; the medication team were not in direct contact with the GP or the patient.

Each medication was examined and compared to the guidelines and tools, as shown in Table 1.

If the patient information contained symptoms suspected of being side effects or prescriptions for side effects (e.g., edema or a prescription of furosemide for edema), we paid special attention to medications known to produce them (e.g., amlodipine). Lastly, we checked whether any diagnoses were lacking treatment. This was summarized in the medication review record with a clear recommendation for each medication. We chose to have four types of recommended changes to the medication: *discontinuation*, *reduction of dose*, *increase of dose* and *change of medication*. *Discontinuation* is the cancellation of the prescription, e.g., because we judged it unnecessary or the patient experienced too many side effects. This change included *discontinuation by tapering*. *reduction of dose* and *increase of dose* both involved an adjustment of the dose, for example, to reach the recommended dose, or on the basis of the laboratory data to meet the goal-of-care. *Change of medication* was a change from one medication to a more suitable one, based on side effects, price or convenience for the patient. This did not include changes between generic drugs, which pharmacies in Denmark are permitted to do.

The medication review record was sent to the medical consultant 7 days before the visit to the GP.

### 2.7. Feedback

During the visit to the GP, the medical consultant presented the recommendations made by the medication team to the GP and discussed each of them. The GP gave feedback on each recommendation and had three possible choices: to agree and accept to make the change; to disagree and decline to make the change; or to be uncertain and not decide at that time to make the recommended change. The GP’s feedback was recorded for later evaluation.

### 2.8. Data Analysis/Statistical Method

Data were managed and analyzed using SAS^®^ software (version 9.4 of the SAS system for Windows. Copyright 2002–2012 ©SAS Institute Inc.). All data were stored using REDCap (Research Electronic Data Capture) [20], which is a secure database approved for that purpose by the Danish Data Protection Agency. No statistical tests or comparisons were made, as this is a descriptive study. Medications, diagnosis, relevant laboratory information, recommended changes and acceptance to recommended changes were described.

From the list of diagnoses and the medical history in the patient cases, we clustered the patients’ diagnoses into 13 disease groups, as listed in Table 2.

### 2.9. Ethics

This project was approved by the Danish Data Protection Agency (I-Suite no 05564). According to Danish law, approval by the Danish Council on Ethics was not required and could not be obtained for this study, as we only recommended changes to the medication. The GPs decided which changes to accept and implement as part of their normal care for the patients. Each patient gave informed consent to be enrolled in the project.

## 3. Results

### 3.1. Patients

We conducted medication reviews of 94 patients with a total of 1471 prescriptions (an average of 15.6 prescriptions per patient). The characteristics of the patients, including their laboratory data, are shown in Table 2.

### 3.2. Prescriptions and Recommendations

**Figure 2:**
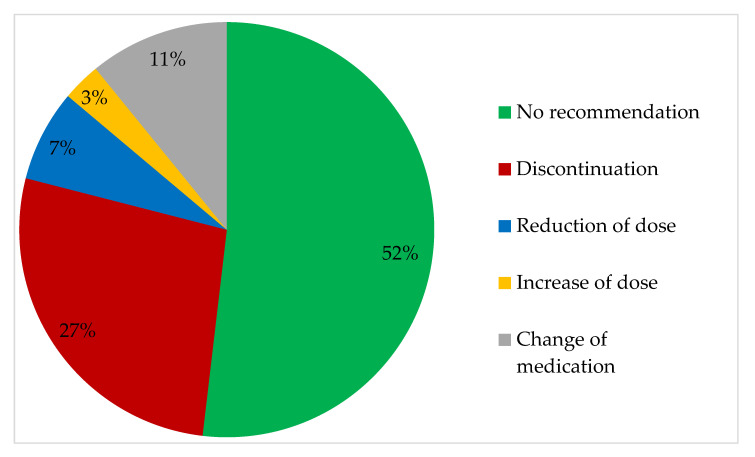
Distribution of the recommendations for the 1471 prescriptions reviewed. For 763 (52%) of the prescriptions no change was recommended.

Of the 1471 prescriptions reviewed, we recommended a change for nearly half (48%) of them, corresponding to 708 recommendations. We recommended at least one change to the medication of all patients. On average we recommended 7.5 changes per patient. The most common of these was *discontinuation*, which accounted for more than half of the recommendations—thereby a quarter of the prescriptions (Figure 2).

**Figure 3:**
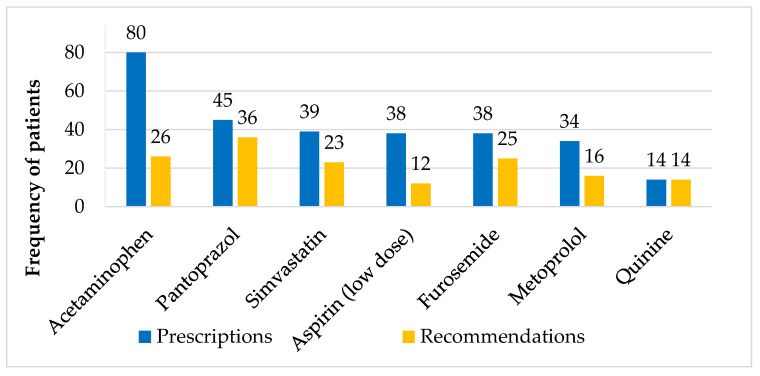
Medications most frequently prescribed or recommended to change. These medications were in the top five most prescribed or most often recommended to change. Data on prescriptions and recommendations are shown for all 94 patients.

The most frequently prescribed medications are set out in Figure 3. Acetaminophen was the most commonly prescribed drug (85% of patients) (also including “as needed” prescriptions). Pantoprazole was the drug most commonly recommended for changes; it was recommended that 38% of patients change their prescription for this medication.

The GPs gave us feedback about our recommendations concerning 580 of the 708 prescriptions (82%). We received feedback from 76 (81%) GPs concerning their acceptance of the recommended changes for their patients. Since each GP had provided information on a single patient, 76 (81%) of patients received feedback about their medication. For these patients, 55% of the recommendations were accepted, 28% were declined; the remaining 17% the GPs were “not sure”. On average, 4.2 recommendations per patient were accepted.

**Figure 4:**
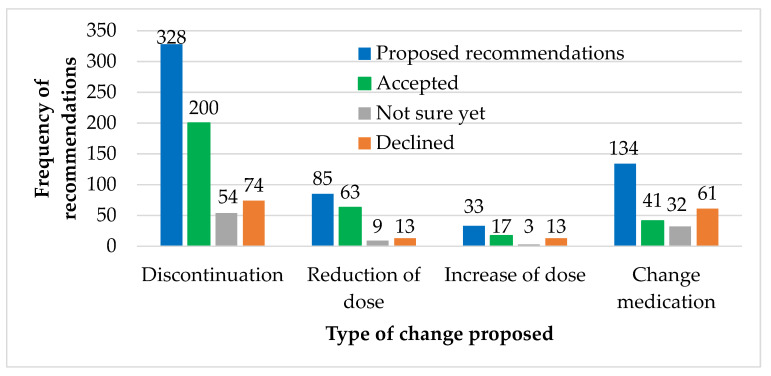
Recommendations with feedback from the general practitioner (n = 580 recommendations about the medications of 76 patients). The prescriptions with recommendations (blue) where the general practitioner had accepted (green), was not sure yet (grey) or declined (orange).

The acceptance rate of *discontinuation* recommendations was 61% (see Figure 4). Half (56%) of the accepted *discontinuation* recommendations were related to medications found in the deprescribing list [19], which is a national list of recommended drug discontinuations. GPs accepted recommendations for 263 prescriptions concerning discontinuation or reduction of dose, making up 18% of all the 1471 prescriptions reviewed. *Reduction of dose* and *change of medication* were the most and least frequently accepted recommendations (74% and 31%), respectively.

### 3.3. Method—Quality of the Patient Cases

We asked the GPs to send a patient case with the required information, as described in the Methods section. In several of the cases received, one or more pieces of information were missing. We expected to receive information on renal function for all patients, as it is considered relevant for this patient group in relation to their medical treatment. However, we received information on renal function, as a written renal assessment or as an eGFR, for 82 of the 94 patients (87.2%). The diagnoses were missing in 28% of the cases, while the indication written next to each medication were missing in 40% and eGFR were missing in 17% of the cases. Neither diagnoses nor indications were available for 7% of the cases.

**Figure 5:**
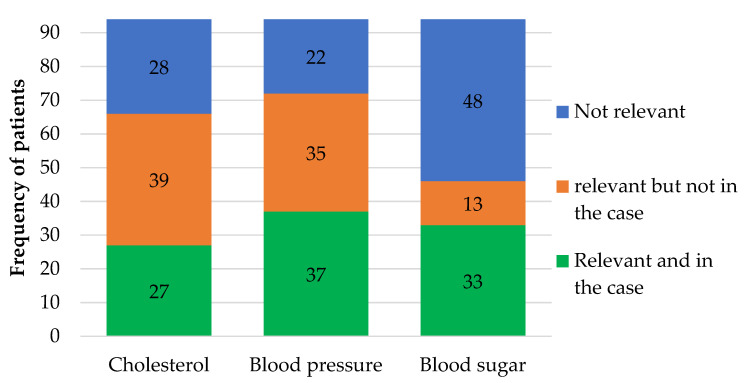
The availability of relevant laboratory data in patient cases. The figure shows the number of patient cases (n = 94) with relevant and available (green) laboratory data, relevant but missing in the patient case (orange) or cases where the laboratory data were not relevant (blue). Laboratory data were considered relevant if the patient had the diagnosis or took medication for hypercholesterolemia, hypertension and diabetes, respectively.

Of the 94 patient cases, one third each were complete, lacking one piece of information and lacking at least two pieces of information. The prevalence of receiving relevant laboratory data for patients with diagnosis or medication for hypercholesterolemia, hypertension or diabetes mellitus is shown in Figure 5.

In order to ensure the high quality of the medication review, additional information was obtained by contacting the GPs. Hence, more diagnoses and laboratory data were used in the medication review than initially included the patient cases.

## 4. Discussion

### 4.1. Primary Findings

In this study, we included medication reviews of 94 patients (1471 prescriptions) from 94 different GPs. We recommended changes for 48% of the prescribed drugs and these were discussed with the patients’ GPs. The GPs accepted *discontinuation* or *reduction of dose* for 18% of all the 1471 prescriptions in this study. This may indicate widespread overmedication, either in the form of unnecessary medication or of excessive doses. These patients were complicated and not representative of the typical polypharmacy patient, as discussed further in Section 4.2.

### 4.2. Recommended Changes to Medication

We assumed that our definition of a recommended change in medication corresponds to outcomes observed in other medication review studies, e.g., drug-related problems and potentially inappropriate prescribing [22,23]. We found an average of 7.5 recommendations per patient; all patients had at least one recommendation. This is higher than the average of 1.1–5.9 drug-related problems found in medication reviews performed jointly by a pharmacist and a GP, described in a literature review by Geurts et al. [3,22,23,24,25,26,27,28,29]. Other medication review studies reported between 84% and 98% of patients to have at least one drug-related problem compared with 100% in this study [22,25,29,30]. The total number of drug-related problems and individuals with at least one drug-related problem depends, of course, on the population being studied. However, when considering studies with inclusion criteria similar to ours, the numbers of drug-related problems are still lower [3,22,23]. This suggests that the high frequency seen in our study is not a consequence of the inclusion criteria. We asked each GP to select one complicated patient. As these patients constituted the most complicated ones managed by each GP, they were prone to be more complicated than the patients considered in similar studies. This was supported by the number of medications recorded per patient (15.6), which was higher than those reported elsewhere (average of 4.6–12.8 medications per patient) [22,23,24,25,26,27,28,29,30,31].

It is important to examine how many recommendations were accepted, as a high frequency of recommendations may be a result of many irrelevant recommendations [32]. We had an acceptance rate of 4.2 recommendations per patient, which is towards the higher end of those noted in similar studies [3,22,23,24,25,26,27,28,29]. The large number of accepted recommendations may indicate that the GPs considered the recommendations relevant, and presumably arises from the consideration of more complicated patients, a thorough medication review with clinically relevant recommendations and face-to-face discussion between the medical consultant and GP.

These accepted recommendations may lead to patients receiving a better overall treatment. Symptom scores or healthcare-related quality of life could be interesting outcomes to examine in future studies, making it possible to investigate whether the patients are better off after the medication review has been performed.

The feedback design of this study complicates comparison with other studies, as the GPs could choose the option “not sure” instead of either of the obvious options of “agreed” and “disagreed”. 17% of the recommendations with feedback were in the “not sure” category. This makes the acceptance rate uncertain, as it could be anywhere between 55%–72% depending on the degree of acceptance of the 17% “not sure” recommendations. To make future studies more comparable with the other studies, it may be preferable not to offer the latter feedback option. However, to give the feedback “agreed” may be misleading when the GP needs to look further into the patient record or to talk to the patient to be sure about the recommended change.

### 4.3. The Method

This method involved at least four healthcare professionals (the GP, the physician and pharmacist from the medication team from the Department of Clinical Pharmacology and the medical consultant from KAP-H), which made it a laborious method with several deadlines for passing on the case or medication review record. These deadlines were hard to meet and were eventually abandoned. However, this structure may yield more rigorous recommendations and a more complete medication review since different healthcare professionals may find different medication-related problems [4]. This method was thorough, not only by the many healthcare professionals inspecting the cases, but also by systematically going through every medication and all paraclinical data, as well as comparing it to relevant guidelines. The method took approximately 5 h per review, not counting the GP’s time. The patients in this study were more complicated and had more medications and more recommended changes than reported in other studies [22,23,24,25,26,27,28,29,30,31]. This may account for and justify the extra resources spent on these medication reviews, as a simpler and less thorough process may not have identified all the relevant recommendations. In our opinion this method of medication review involved too many health professionals and too many challenges to obtaining the mandatory information from general practice, and consequently was too time-consuming. Therefore, we see little value in adopting this exact method in the future, although another method may be needed to support general practice.

One approach to reducing the time required in future projects could be the use of simple screening, e.g., comparing the patients’ medication list with the medications mentioned in the deprescribing list [19]. The medications that were most often recommended for change in this study are already known to cause drug-related problems (e.g., furosemide for a patient without heart failure). The deprescribing list contains all the medications most frequently prescribed in this study except for acetaminophen and metoprolol, which we will propose should be added. After completion of this project, acetaminophen was added to the deprescribing list. The 56% of accepted *Discontinuation* recommendations regarding medications that are mentioned in the deprescribing list [19] would have been identified by quick and simple screening, leaving a substantial 44% of recommendations unidentified. Even though simple screening could reduce polypharmacy, it would not be able to help patients with more complex medication regimes, nor would it be able to replace a medication review. However, it could be done in advance to make the medication review easier—or as a faster and cheaper way to reduce polypharmacy in general practices.

Another way to simplify the structure would be to reduce the number of healthcare professionals involved, for example to a maximum of two, which both conduct the medication review and visits the GP.

### 4.4. Patient Case

In this project we requested laboratory data from the GPs because Freeman et al. showed that a pharmacist with access to laboratory data and medical history makes a higher degree of relevant recommendations [32]. Likewise, Hurkens et al. demonstrated how healthcare professionals made more correct recommendations during medication review when more data were available [4]. As the results show, several patients had laboratory values outside the normal range of goal-of-care. This resulted in recommended changes to the medication that would have been overlooked if the laboratory data had not been included. The laboratory data thus made the medication review more clinically relevant. In some cases, the GP may not have been aware of the goal-of-care, especially for those that are individualized, e.g., blood pressure, blood sugar and cholesterol. As an example, the goal-of-care for blood sugar changes markedly with age. In the first years after diagnosis, the goal-of-care is HbA1c < 48 mmol/mol, but this changes over time so that in old age, when the patient has a higher risk of hypoglycemia and a lower risk of complications occurring or emerging before death, the goal-of-care may be to avoid symptoms with an HbA1c < 76 mmol/mol [9].

The lack of handover of complete patient cases from the GPs was a major limitation of our method, causing some diagnoses to be derived from the indications automatically specified from the medication list. This meant that the medication review was of lower quality because some drug-related problems, e.g., medication prescribed without associated diagnoses, may not have been detected. This challenge was even more serious for medication lists without indications. In these circumstances, an indication may have been presumed by the medication team in order to evaluate the medication. Therefore, the medication may be considered relevant, leading to dangerous circular reasoning. One solution for incomplete cases in future studies would be to gain access to the GPs’ record systems. This would be more time-consuming for the healthcare professionals conducting the medication review, but less so for the patients’ GPs. The incorporation of a pharmacist into general practice has been implemented in England and a model with a pharmacist in general practice was tried in Denmark in the early 2000s, but was never implemented [28,33]. The time may be ripe to try a similar model again. Another solution would be to access the data when visiting the GPs, but this would require more of the GPs time during the visits.

## 5. Conclusions

There is a need for medication reviews for patients in general practice. Many patients have prescriptions that are unnecessary or feature excessively high doses; these can be identified through a medication review similar to that described here. With this new method, we found high frequencies of recommendations per patient and of accepted recommendations per patient. Unfortunately, the basis for performing the medication review was generally not complete as some essential patient information was missing. This highlights an area that could be improved in future medication reviews. Our method proved to be very time-consuming and complex for the several healthcare professionals involved. Therefore, we do not see much prospect of the widespread implementation of this exact method. A medication review of similar thoroughness may nevertheless be useful for the most complicated and heavily medicated patients. A new method could advantageously include laboratory data and an interdisciplinary medication team with the focus on the direct contact between the medication team and the GPs, to avoid an overly complex structure.

## Figures and Tables

**Table 1:** Each medication on the medication list was examined according to the criteria listed in the medication review.

Examination of Whether Provision of the Medication is Rational on the Basis of the Following Criteria	Comparison with the Following Guidelines/References
Indication matching diagnoses	Guidelines on specific diseases from Danish or international professional associations [11,12,13,14,15,16,17]
Contraindication	
	Basislisten (The list of recommended medication in the Capital Region of Denmark) [18]
Dose	
	The Deprescribing List [19] of medications that can often be discontinued, including information on when and how to discontinue. This is published jointly by the Danish Health Authority and the regions of Denmark.
Form of dosage	
	Drug-drug interactions using two interaction databases (http://interaktionsdatabasen.dk and http://www.micromedexsolutions.com)
Time of dosage	
Price	

**Table 2:** Patient characteristics. For laboratory data and eGFR, we did not receive information on all patients.

**Information**	**Median**	**Interquartile Range**	**Range**	**Patients (n)**
Age (years)	79	72–83	65–98	94
eGFR (mL/min)	63	46–80	19–92	82
**Sex**		**Percentage of study population**		**Patients (n)**
Female		56.4		53
Male		43.6		41
**Stated diagnosis**		**Percentage of study population**		**Patients (n)**
Cardiovascular		90		85
Musculoskeletal		68		64
Lung		52		49
Diabetes		50		47
Other		45		42
Gastrointestinal		33		31
Psychiatric		26		24
Pain		24		23
Metabolism		20		19
Neurologic		19		18
Cancer		16		15
Kidney		13		12
Infection		3		3
**Laboratory data**	**Mean**	**Min-max**	**Goal-of-care**	**Patients (n)**
Blood sugar HbA1c (mmol/mol)	50.32	30.00–82.00	Individual < 48–75 [9]	49
Cholesterol LDL (mmol/l)	2.33	0.2–6.7	Individual < 1.8–3.0 [21]	52
Blood pressure (mmHg)	136/74	100/40–180/97	Individual < 130/80–145/85 [10]	61

Abbreviations: eGFR (estimated glomerular filtration rate), HbA1c (glycated hemoglobin), LDL (low-density dipoprotein).
